# Fast MR Imaging of the Paediatric Abdomen with CAIPIRINHA-Accelerated T1w 3D FLASH and with High-Resolution T2w HASTE: A Study on Image Quality

**DOI:** 10.1155/2015/693654

**Published:** 2015-04-07

**Authors:** Mengxia Li, Beate Winkler, Thomas Pabst, Thorsten Bley, Herbert Köstler, Henning Neubauer

**Affiliations:** ^1^Department of Radiation Oncology, University Hospital Wuerzburg, 97080 Würzburg, Germany; ^2^Department of Paediatrics, University Hospital Wuerzburg, 97080 Würzburg, Germany; ^3^Department of Diagnostic and Interventional Radiology, University Hospital Wuerzburg, 97080 Würzburg, Germany

## Abstract

The aim of this study was to explore the applicability of fast MR techniques to routine paediatric abdominopelvic MRI at 1.5 Tesla. “Controlled Aliasing in Parallel Imaging Results in Higher Acceleration-” (CAIPIRINHA-) accelerated contrast-enhanced-T1w 3D FLASH imaging was compared to standard T1w 2D FLASH imaging with breath-holding in 40 paediatric patients and to respiratory-triggered T1w TSE imaging in 10 sedated young children. In 20 nonsedated patients, we compared T2w TIRM to fat-saturated T2w HASTE imaging. Two observers performed an independent and blinded assessment of overall image quality. Acquisition time was reduced by the factor of 15 with CAIPIRINHA-accelerated T1w FLASH and by 7 with T2w HASTE. With CAIPIRINHA and with HASTE, there were significantly less motion artefacts in nonsedated patients. In sedated patients, respiratory-triggered T1w imaging in general showed better image quality. However, satisfactory image quality was achieved with CAIPIRINHA in two sedated patients where respiratory triggering failed. In summary, fast scanning with CAIPIRINHA and HASTE presents a reliable high quality alternative to standard sequences in paediatric abdominal MRI. Paediatric patients, in particular, benefit greatly from fast image acquisition with less breath-hold cycles or shorter sedation.

## 1. Introduction

Image quality and image acquisition time are crucial factors in paediatric abdominal MR imaging. High quality scans with excellent spatial resolution, and correspondingly lengthy scan times, are often needed to identify and assess anatomical structures and pathological lesions within the small scale of paediatric anatomy. Repeated breath-holds and prolonged immobilization within the MR scanner, however, are poorly tolerated by most young patients. Sedation or deep anaesthesia with intubation is thus frequently required in patients of, and occasionally beyond, preschool age [1].

With the latest generation of MR scanner hardware and software, novel scanning techniques have become available for clinical routine imaging that may help to successfully address these challenges. Image acquisition in free-breathing mode with automated motion correction avoids repeated breath-hold cycles while maintaining image quality and spatial resolution [2]. Other techniques focus on greatly accelerated image acquisition beyond the scope of conventional parallel imaging techniques and employ novel modes of data sampling with *k*-space radial data read-out or with data undersampling [3]. Among the latter, T1w imaging accelerated with the Controlled Aliasing in Parallel Imaging Results in Higher Acceleration (CAIPIRINHA) technique produces high quality images with very short acquisition time in volunteers and adult patients [4–7]. Furthermore, with modern scanner and coil hardware, previously available acceleration techniques, such as Half-Fourier Acquisition Single-Shot Turbo Spin-Echo (HASTE) for T2-weighted imaging, now yield significantly improved image quality and may have become a suitable substitute for slower scanning techniques. A scan protocol combining these fast scanning techniques and diffusion-weighted imaging may allow a full abdominal MRI study within 10-minute acquisition time or even less.

In our study, we evaluated the image quality of CAIPIRINHA-accelerated T1w 3D fast low angle shot (FLASH) imaging and fat-saturated T2w HASTE imaging in comparison to standard sequences in a group of paediatric patients undergoing abdominopelvic MRI.

## 2. Patients and Methods

We retrospectively identified a cohort of fifty consecutive patients (age 10 ± 6 years, range 2 years to 18 years, 27 females) who had had routine abdominal MRI at 1.5 Tesla (Magnetom AERA, VD 13, Siemens Medical, Erlangen, Germany). All patients were in good general condition, had normal renal function, and underwent clinically indicated MRI examinations for tumour staging (*n* = 9), tumour follow-up (*n* = 33), and further diagnostic work-up after inconclusive findings on ultrasonography (*n* = 8). Informed written consent was obtained from the legal guardians and, if possible, from the patients. All study work was conducted in accordance with the Declaration of Helsinki (1964).

All patients were examined head first in supine position with phased-array body coils and an intravenous line in place. Sedation was administered and continuously supervised by an experienced paediatric anaesthesiologist. The scanning protocol comprised T2w, T1w precontrast, T1w contrast-enhanced, and diffusion-weighted imaging, as clinically necessary. Postcontrast scans were acquired after i.v. administration of a weight-adapted standard dose of gadoterate meglumine (Dotarem, Guerbet). Details of typical scan parameters are outlined in Table 1. Some parameters, such as field of view or slice thickness, were adjusted depending on patient size, as clinically necessary. All scans included transverse CAIPIRINHA-accelerated contrast-enhanced- (ce-) T1w 3D FLASH imaging with fat saturation (FS). Standard of comparison was ce-T1w 2D FLASH FS imaging in 40 nonsedated patients and respiratory-triggered ce-T1w TSE FS imaging in 10 sedated young patients. In nonsedated patients, CAIPIRINHA 3D FLASH and standard 2D FLASH scans were acquired in random order, while CAIPIRINHA always followed T1 TSE imaging in sedated patients. Twenty nonsedated patients had both coronal T2w Turbo Inversion Recovery Magnitude (TIRM) imaging and high-resolution fat-saturated T2w HASTE imaging prior to i.v. contrast administration.

Two readers, a resident with basic training in MR imaging and a board-certified consultant paediatric radiologist with 10-year experience, each performed one independent blinded reading, followed by a consensus reading. Prior to the study reading, both readers reviewed a training set of 10 patients (not included in the study) in consensus to arrive at a common diagnostic standard. Assessment was based on the presence or absence of diagnostic image quality and the presence of artefacts. Overall image quality of CAIPIRINHA versus FLASH/TSE and HASTE versus TIRM was graded on a modified 5-item Likert scale with the categories: 1 = insufficient for diagnosis, 2 = degraded image quality, still sufficient for diagnosis, 3 = satisfactory image quality despite present artefacts, 4 = good image quality, some artefacts, and 5 = excellent image quality, little or no artefacts. All readings were performed on a certified work station (Syngo Plaza, Siemens Medical, Erlangen).

### 2.1. Statistical Analysis

Patient data are given as mean and standard deviation. Sample size for the comparison of CAIPIRINHA versus FLASH was estimated with the software G^∗^Power Version 3.1.9. [7] as *n* = 38, based on a training data set of 10 patients (not included in the study) and the parameters *α* = 0.05, power = 0.95, and effect size 0.56. Kappa statistics according to the Landis and Koch schema were used to compare the interobserver agreement on image quality [8]. Image quality between study sequence and standard sequence was compared using the Wilcoxon matched-pair signed-rank test. Proportions on cross tabs were tested with the Chi-square test. All statistical tests were performed as two-sided tests with *α* = 0.05 and were computed with the IBM SPSS 21 software package for Windows.

## 3. Results

Analysis of interobserver agreement showed a Cohen's kappa value of 0.65 across all readings, indicating substantial agreement. The grading between the two readers differed by not more than one category in all cases of disagreement. The results of the consensus reading were used for all further analyses.

### 3.1. Standard FLASH versus CAIPIRINHA-Accelerated T1w Imaging

**Figure 1:**
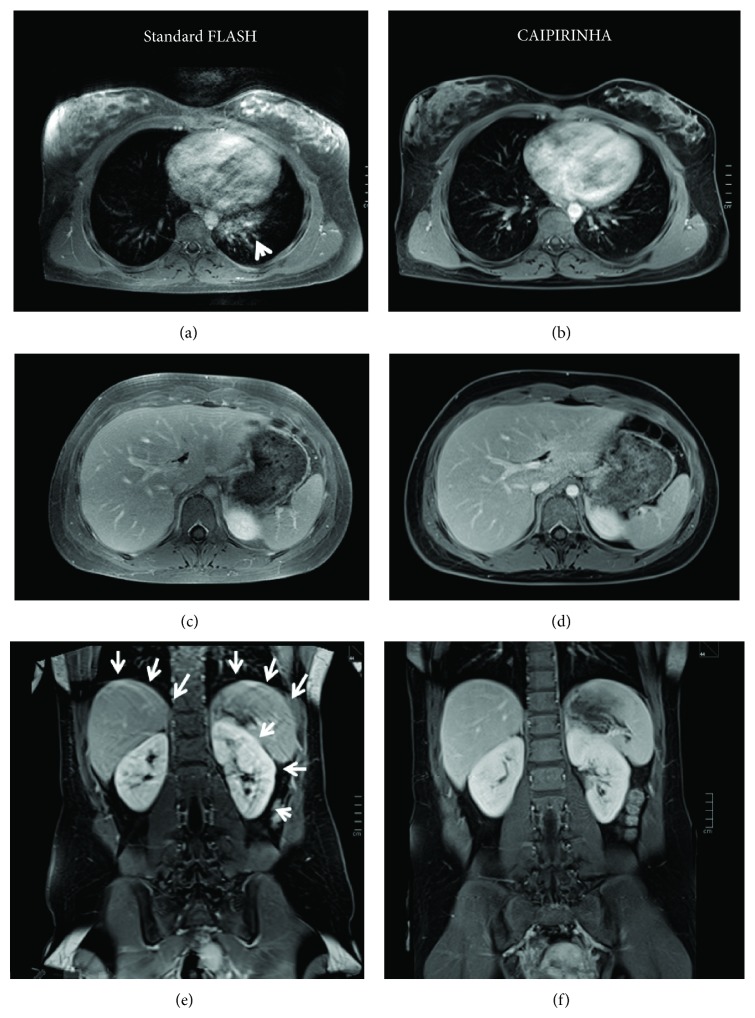
Thoracic and abdominal MRI study in a 17-year-old female patient with Hodgkin's disease in complete remission. Images are displayed for contrast-enhanced fat-saturated standard FLASH (a, c, e) and CAIPIRINHA-accelerated T1w 3D FLASH (b, d, f) from the transverse thoracic image stack (a, b), the transverse abdominal image stack (c, d), and the coronal abdominal image stack (e, f). The sequences were scanned in turns on the respective level as standard FLASH first, followed by CAIPIRINHA. With CAIPIRINHA, there are markedly less retrocardial artefacts ((b), as compared to standard FLASH (a), arrowhead). While the transverse thoracoabdominal scans otherwise show excellent image quality with both FLASH and CAIPIRINHA, the patient could eventually no longer fully comply with the frequent breath-holds, so that the coronal FLASH image (e, arrows) is degraded by respiratory motion artefacts and shows blurring of contours along the diaphragm and the kidneys. The final acquisition of the coronal CAIPIRINHA sequence (f) again is virtually free of artefacts. The transverse abdominal scans were included in the study analysis and were consistently graded as IQ = 5 by both readers.

All image sets showed diagnostic image quality (IQ); that is, IQ level > 1. Median image quality was 4 with FLASH and 5 with CAIPIRINHA with a range of 2 to 5 and 3 to 5, respectively (Table 2). The quality score was higher for CAIPIRINHA studies, compared to FLASH (Wilcoxon matched-pair signed-rank test, *P* < 0.001) (Figure 1). However, the proportion of satisfying to excellent image quality (quality levels 3 to 5) of CAIPIRINHA imaging did not differ from standard FLASH technique (Chi-square test, *P* > 0.05).

### 3.2. Triggered T1 TSE versus CAIPIRINHA-Accelerated T1w Imaging in Sedated Patients

Image quality was excellent in 6 of 10 patients with respiratory-triggered T1 TSE (Table 2). Two more patients showed suboptimal image quality with standard imaging, while irregular breathing of the sedated child partially disabled respiratory triggering in two cases and resulted in severe respiratory motion artefacts. In these two patients, CAIPIRINHA produced satisfactory image quality. Image quality in general tended to be better with triggered TSE imaging (*P* = 0.08). In spite of fast image acquisition, marked respiratory motion artefacts were present with CAIPIRINHA in the majority of patients. Navigator-triggered scans resulted in a higher proportion of excellent and good image quality, compared to CAIPIRINHA (*P* < 0.05), whereas the proportion of satisfying to excellent image quality did not differ significantly (*P* > 0.05).

### 3.3. T2w TIRM versus T2w HASTE FS

**Figure 2:**
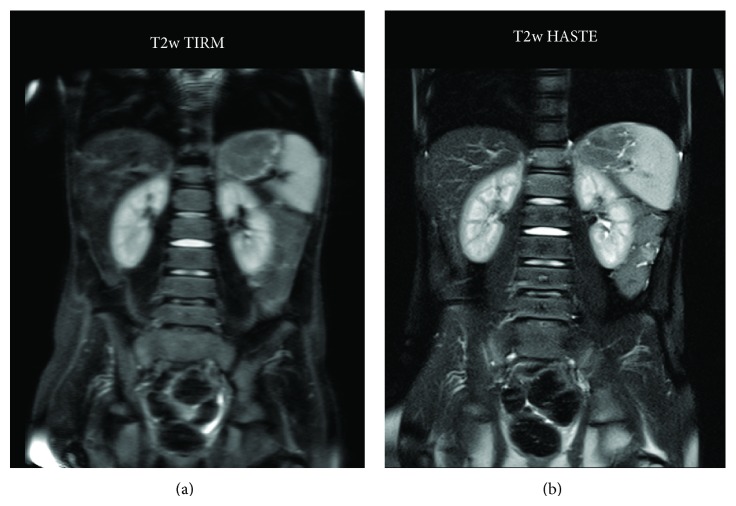
Coronal T2w TIRM (a) and high-resolution T2w HASTE FS (b) imaging in an 8-year-old girl with chronic abdominal pain and normal abdominal ultrasonography. In the absence of any pathological findings, the TIRM image, owing to the long repetition time, is considerably blurred by motion artefacts in the restless and continuously shifting patient. Repeating the T2 TIRM sequence did not improve image quality. HASTE imaging does not show any motion artefacts and clearly depicts bony structures including the lower thoracic and lumbal spine as well as the abdominal organs. Incomplete saturation of fatty tissue signal towards lower image margin is more noticeable with HASTE than with TIRM imaging. Image quality was graded as IQ = 2 (TIRM) and IQ = 4 (HASTE).

Overall image quality was higher with HASTE imaging, compared to TIRM (*P* = 0.001) (Table 2). All image sets, except for two TIRM studies, showed at least satisfactory image quality (Figure 2). While in most patients both TIRM and HASTE imaging clearly visualised spinal and paraspinal anatomical structures, the delineation of upper abdominal organs was less frequently compromised by artefacts with HASTE FS.

## 4. Discussion

Our study results demonstrate that abdominal MR imaging with CAIPIRINHA and HASTE technique facilitates fast scans with similar, or superior, image quality, compared to standard scans.

Recent years have seen a surge in abdominal MR imaging studies among both adult and paediatric patients for its merits of superior soft tissue contrast and image acquisition without ionising radiation. However, abdominal MR scans still are challenging, especially for children, as repeated breath-holding and prolonged immobilisation in the scanner are usually required [9]. Modern scanning techniques substantially accelerate MR image acquisition. In our study, acceleration of T1w postcontrast imaging with CAIPIRINHA reduced scanning time of one full set of transverse abdominal T1w images from about four minutes with eight 20-second breath-holds to a single breath-hold of 15 seconds, thus approaching the speed of computed tomography imaging. Our data indicate that there is no trade-off in terms of image quality. Rather, on the contrary, image quality seems to improve with faster scans as a result of better patient compliance and less motion artefacts. All paediatric patients in our study underwent scheduled routine examinations; most of them were in good general clinical condition and also had experienced MRI examinations before. Compared to our study group, the superiority of fast scanning would presumably be even more pronounced in patients with reduced clinical condition or in emergency imaging.

Various techniques accelerating MR image acquisition have been developed to improve image quality and to reduce scanning time. Recent innovations focus on non-Cartesian acquisition of *k*-space data, such as PROPELLER or BLADE [9, 10], single-shot image acquisition, such as half-Fourier rapid acquisition with relaxation (RARE), HASTE, or echo-planar imaging (EPI), and *k*-space undersampling, such as compressed sensing [11] or CAIPIRINHA [4]. CAIPIRINHA is an extension of the Generalized Autocalibrating Partially Parallel Acquisition (GRAPPA) technique [4, 5]. It exploits sensitivity variations in the receiver coil array more efficiently and thus allows higher acceleration factors without loss of image quality. Abdominal CAIPIRINHA-accelerated T1w scans have been studied in adult volunteers and patients in comparison to standard sequences [5, 12, 13]. Reported data on CAIPIRINHA compares well to standard sequences. Image quality with CAIPIRINHA was superior when standard sequences were used with acceleration factors equalling CAIPIRINHA's acceleration [5]. In a study by Wright et al., experienced readers preferred CAIPIRINHA images to GRAPPA standard images in more than two-thirds of the image pairs [13]. Another recent study investigated CAIPIRINHA in combination with different gadolinium-containing contrast agents (gadobutrol, gadoterate meglumine, and gadoxetic acid) and found good image quality for all substances without significant differences [14]. So far, there is no published data available on CAIPIRINHA-accelerated imaging in paediatric patient populations. Our findings provide first evidence that acceleration with CAIPIRINHA does not adversely affect image quality in paediatric abdominal scans. Instead, CAIPIRINHA proved to be the more reliable and robust imaging technique.

In patients undergoing MRI with sedation, respiratory-triggered T1w TSE yielded the best imaging results. Depending on the particular breath cycle in each patient, scanning time ranged from 6 to more than 10 minutes. In two of our patients, triggering was not fully effective resulting in severe motion artefacts. In these patients, CAIPIRINHA imaging actually produced better image quality. Otherwise, CAIPIRINHA could not compensate for the fast breathing rate typical of young paediatric patients and did not appear advantageous in sedated free-breathing patients. At present, CAIPIRINHA may be a fast alternative if respiratory triggering fails. In the future, CAIPIRINHA may help to shorten sedation if combined with triggering or other techniques of motion compensation.

Single-shot T2w imaging with HASTE image acquisition has been available for years and is widely used for imaging of the biliary system (MRCP) and as a quick-to-scan preliminary scout sequence. Yet with the latest generation of MR scanners, image quality has improved considerably, so that now T2w HASTE may serve as a fast diagnostic imaging sequence. Paediatric abdominal scan protocols at our institution often include a T2w scan with fat saturation, usually a coronal T2w TIRM scan, for detection of tumorous or inflammatory foci in the spine or abdominal wall. In our study, fat-saturated T2w HASTE images showed better overall image quality and less motion artefacts, compared to T2w TIRM. In our experience, HASTE cannot yet fully match the image quality of TIRM imaging under optimal conditions. In paediatric imaging, however, we seldom enjoy such optimal conditions, and therefore, in our clinical cohort, HASTE was a valuable tool and was found superior to TIRM in considerable proportion of patients. HASTE imaging is more susceptible to inhomogeneous fat saturation in peripheral field of view. While TIRM imaging applies an inversion high frequency pulse to null the fat signal, we used standard spectral fat saturation with our HASTE sequence. Other techniques of fat saturation, such as Spectral Attenuated Inversion Recovery (SPAIR) or the Dixon technique, may alleviate the issue of incomplete fat saturation with HASTE in the future.

Two limitations of our study warrant discussion. First of all, the investigated MR sequences all come with their characteristic image impression, which defeats any attempt to blind the experienced reader to the particular sequence. Therefore, a personal preference of the readers may have influenced study results. This, however, is a general problem and holds true for most, if not all, studies comparing quality and imaging characteristics across different scanning techniques. Secondly, the number of focal lesions in our present patient cohort was not sufficient to perform a quality assessment for conspicuity evaluation of focal pathologies. Previous studies on CAIPIRINHA demonstrated robust pancreatic imaging and better delineation of organ outlines with CAIPIRINHA, as compared to GRAPPA-accelerated 2D FLASH imaging [12], better identification of stalk and posterior lobe of the pituitary gland with dynamic CAIPIRINHA-accelerated scans [15], and better vessel clarity, lesion conspicuity, and edge sharpness in liver scans of adult patients [6]. From our clinical experience we know that HASTE imaging with its fast “one-slice-per-shot” acquisition in combination with breathing-related motion in the upper abdomen can lead to incomplete volume coverage, so that, for example, small liver lesion may be missed. On the other hand, HASTE may reveal focal lesions which are not detectable on TIRM due to motion artefacts. One may hypothesize that in general fast scanning techniques may allow better detection of focal abdominal lesions, if only because of less imaging artefacts. Still, conspicuity of focal lesions with CAIPIRINHA and with HASTE deserves further evaluation and will be addressed in future research.

In summary, CAIPIRINHA-accelerated T1w imaging and high-resolution HASTE present innovative imaging techniques in paediatric abdominal MRI and facilitate high quality imaging at very short scanning times. Fast MRI scans with fewer breath-holds improve patient compliance. Thus, abdominal MRI without the need of sedation may become feasible for a larger proportion of preschool and young school children. Employing HASTE, CAIPIRINHA, and diffusion-weighted sequences, a comprehensive abdominal MRI scan is now possible with less than 10 minutes of acquisition time.

## 5. Conclusion

CAIPIRINHA and HASTE techniques greatly accelerate paediatric abdominal MRI and present a fast and reliable alternative to standard MR imaging sequences. Fast image acquisition improves patient comfort and patient compliance and thus contributes to better image quality and less invasiveness of paediatric MRI.

## Figures and Tables

**Table 1:** Typical MRI scan parameters, as used in this study.

	Standard 2D FLASH	CAIPIRINHA 3D FLASH	T1w TSE triggered	T2w TIRM	T2w HASTE FS
TR, ms	86.0	6.1	2000	8290	1000
TE, ms	3.8	3.0	2.5	84	102
Flip angle, °	90	10	15	180	160
Scan orientation	Transverse	Transverse	Transverse	Coronal	Coronal
In-plane resolution, mm	1.3 × 1.3	1.0 × 1.0	0.7 × 0.7	0.8 × 0.8	1.0 × 1.0
Slice thickness, mm	5	3–5	4	4	4
FOV, mm	250	250	350	350	350
iPAT	None	Acceleration factor = 4	None	Acceleration factor = 2	Acceleration factor = 2
Breath-hold	22 s	15 s	Respiratory-triggered	20 s	21 s
Scan time	4 min 3 s	15 s	6 min 10 s	3 min 5 s	42 s

TR: repetition time, TE: echo time, FOV: field of view, and iPAT: Integrated Parallel Acquisition Techniques.

**Table 2:** Results of the consensus reading on overall image quality.

	Standard 2D FLASH *n* = 40	CAIPIRINHA 3D FLASH *n* = 40	T1w TSE sedation *n* = 10	CAIPIRINHA sedation *n* = 10	T2w TIRM *n* = 20	T2w HASTE FS *n* = 20
IQ 5	12	22	6	2	8	14
IQ 4	25	17	1	2	8	5
IQ 3	2	1	1	6	2	1
IQ 2	1	—	2	—	2	—
IQ 1	—	—	—	—	—	—

IQ: image quality level, ranging from 1 = insufficient for diagnosis to 5 = excellent.

## References

[1] Sury M. R. J., Smith J. H. (2008). Deep sedation and minimal anesthesia. *Paediatric Anaesthesia*.

[2] Zhang T., Cheng J. Y., Potnick A. G. (2015). Fast pediatric 3D free-breathing abdominal dynamic contrast enhanced MRI with high spatiotemporal resolution. *Journal of Magnetic Resonance Imaging*.

[3] Kim B. S., Lee K. R., Goh M. J. (2014). New imaging strategies using a motion-resistant liver sequence in uncooperative patients. *BioMed Research International*.

[4] Breuer F. A., Blaimer M., Mueller M. F. (2006). Controlled aliasing in volumetric parallel imaging (2D CAIPIRINHA). *Magnetic Resonance in Medicine*.

[5] Riffel P., Attenberger U. I., Kannengiesser S. (2013). Highly accelerated T1-weighted abdominal imaging using 2-dimensional controlled aliasing in parallel imaging results in higher acceleration: a comparison with generalized autocalibrating partially parallel acquisitions parallel imaging. *Investigative Radiology*.

[6] Yu M. H., Lee J. M., Yoon J.-H., Kiefer B., Han J. K., Choi B.-I. (2013). Clinical application of controlled aliasing in parallel imaging results in a higher acceleration (CAIPIRINHA)-volumetric interpolated breathhold (VIBE) sequence for gadoxetic acid-enhanced liver MR imaging. *Journal of Magnetic Resonance Imaging*.

[7] Park Y. S., Lee C. H., Kim I. S. (2014). Usefulness of controlled aliasing in parallel imaging results in higher acceleration in gadoxetic acid-enhanced liver magnetic resonance imaging to clarify the hepatic arterial phase. *Investigative Radiology*.

[8] Landis J. R., Koch G. G. (1977). The measurement of observer agreement for categorical data. *Biometrics*.

[9] Darge K., Anupindi S. A., Jaramillo D. (2011). MR imaging of the abdomen and pelvis in infants, children, and adolescents. *Radiology*.

[10] Hirokawa Y., Isoda H., Maetani Y. S., Arizono S., Shimada K., Togashi K. (2008). MRI artifact reduction and quality improvement in the upper abdomen with PROPELLER and Prospective Acquisition Correction (PACE) Technique. *The American Journal of Roentgenology*.

[11] Wech T., Pickl W., Tran-Gia J. (2014). Whole-heart cine MRI in a single breath-hold—a compressed sensing accelerated 3D acquisition technique for assessment of cardiac function. *Rofo*.

[12] Haneder S., Koziel K., Morelli J. N. (2014). Clinical application of 3D VIBECAIPI-DIXON for non-enhanced imaging of the pancreas compared to a standard 2D fat-saturated FLASH. *Clinical Imaging*.

[13] Wright K. L., Harrell M. W., Jesberger J. A. (2014). Clinical evaluation of CAIPIRINHA: comparison against a GRAPPA standard. *Journal of Magnetic Resonance Imaging*.

[14] Budjan J., Ong M., Riffel P. (2014). CAIPIRINHA-Dixon-TWIST (CDT)-volume-interpolated breath-hold examination (VIBE) for dynamic liver imaging: comparison of gadoterate meglumine, gadobutrol and gadoxetic acid. *European Journal of Radiology*.

[15] Fushimi Y., Okada T., Kanagaki M. (2014). 3D dynamic pituitary MR imaging with CAIPIRINHA: initial experience and comparison with 2D dynamic MR imaging. *European Journal of Radiology*.

